# The Effect of Wood Ash and Soil Applications on the Behavior and Survival of *Spodoptera frugiperda* (Lepidoptera: Noctuidae) Larvae on Maize

**DOI:** 10.3390/insects14100813

**Published:** 2023-10-13

**Authors:** Nozibusiso Maphumulo, Hannalene du Plessis, Johnnie Van den Berg

**Affiliations:** Unit for Environmental Sciences and Management, North-West University, Potchefstroom 2520, South Africa

**Keywords:** maize pest management, alternative control methods, integrated pest management, smallholder farmers

## Abstract

**Simple Summary:**

Fall armyworm is difficult to control, due to the cryptic feeding of larvae deep inside maize whorls. Even insecticide spray applications do not always effectively reach larvae inside maize whorls. Applying sand/soil directly into whorls of maize plants once an infestation is noticed, is highly relevant in smallholder farming contexts. However, since the efficacy of these methods has not been evaluated before, the need exists to assess their efficacy and to determine application rates. This study was conducted under greenhouse and laboratory conditions on maize plants artificially infested with larvae of different instars. The efficacy of treatments was generally low and varied largely between experiments. Ash treatments resulted in significant mortality of 1st and 5th instars under laboratory conditions. Despite variability in efficacy, these alternative methods have value in IPM systems for smallholder farmers, provided they are applied timeously.

**Abstract:**

Cryptic feeding inside maize whorls makes it difficult to control fall armyworm (FAW). Smallholder farmers use alternative methods of control, of which the efficacy is uncertain. We determined the efficacy of wood ash and soil for the control of FAW and recorded its effect on larval preference and ballooning. Maize plants were artificially infested with larvae of different instars and treatments were either soil, wet ash, or dry ash, applied as single preventative or curative applications. Larvae exhibited non-preference for treated leaves in choice tests. The efficacy of treatments varied largely between experiments. Under laboratory conditions, ash treatments resulted in significant mortality of 1st and 5th instars. Dry and wet ash as curative applications for 1st instars resulted in 67 and 66% mortality, respectively, compared to mortality recorded in the control (22%). Under field conditions, survival of 3rd instars on treated plants was low (21–34%), compared to 70% on untreated plants. Due to the high variability in efficacy, the use of these alternative methods does not guarantee effective control. They do, however, have a place in IPM systems if applied as soon as infestations are observed and when larvae as still small. Recommendations on the use of ash and soil as spot treatments against FAW are provided.

## 1. Introduction

Fall armyworm (FAW), *Spodoptera frugiperda* (J.E. Smith) (Lepidoptera: Noctuidae), is one of the most important pests of maize in Sub-Sahara Africa, where approximately 95% of the area under maize cultivation is deemed climatically suitable for this pest [1]. High economic losses due to this pest have been reported in many African countries [2,3,4], but yield losses may often be over-estimated [5,6]. 

Fall armyworm is difficult to control, due to the cryptic feeding of larvae deep inside maize whorls. Even insecticide spray applications do not always effectively reach larvae inside maize whorls and efficacy may be further reduced if infestations are detected too late [7,8,9,10].

Although insecticide application is the most common method of control [10,11,12,13], many smallholder farmers also use alternative methods such as hand picking, pesticidal plants, or application of wood ash and soil [13,14,15,16,17,18,19,20,21,22]. Applying sand/soil directly into whorls of maize plants once an infestation is noticed is highly relevant in smallholder farming contexts and has long been practiced in Africa [12,23,24], the Americas [25,26], and Sri Lanka [27]. In Zimbabwe, some farmers apply ash (10 g/plant) into the whorl of each maize plant on their fields at weekly intervals from five weeks after planting until tasseling, as well as onto maize ears once they start to develop [18]. Applications of ash at such a high frequency and regular intervals could, to some extent, be considered as a preventative measure.

Ash and soil, either on their own or in mixtures with pesticides, are also used for the control of lepidopteran maize stem borers [28,29] and *S. frugiperda* [22,25,30]. In most cases, the ash used for the control of pests is collected from household kitchens or burnt wood [22,28]. However, since the level of control provided by these alternative methods may be highly variable or not effective [26], the need exists to evaluate their efficacy [16,17,23,31], and to determine application rates [22,28,32]. The aims of this study were to determine the efficacy of wood ash and soil applied into maize whorls for control of *S. frugiperda*, and to study the effect of these applications on behavioral aspects such as larval preference and ballooning, which may further contribute to decreased larval numbers on plants.

## 2. Materials and Methods

### 2.1. Rearing of Fall Armyworm Larvae

FAW larvae were collected in maize fields at Groblersdal (S 25°16′28.21″; E 29°25′23.74″) in the Limpopo Province of South Africa. The larvae were maintained at the Entomology Laboratory of North-West University, where they were reared on Stonefly Heliothis artificial diet (Ward’s Natural Science Establishment, LLC (Rochester, NY, United States) and maize whorl leaf tissue until they pupated. Pupae were collected and individually transferred into 50 mL containers covered with mesh lids to allow for airflow. Upon emergence, male and female moths were paired and put into plastic containers (38 cm × 27 cm × 14.5 cm) covered with mesh lids. Wax paper was inserted inside the containers as an oviposition substrate. A 10% sugar solution was provided in cotton buds as a source of energy for moths. Egg batches were collected daily and transferred into 50 mL containers until they hatched. After the eggs hatched, the neonate larvae were transferred into 500 mL plastic rearing containers containing artificial diet or maize whorl tissue leaves. Diet was provided to small larvae for a period of approximately 10 days, after which leaf tissue was provided for final instars until they started to pupate. Containers were covered with mesh lids and larvae were reared under constant climate conditions at a temperature of 28 °C, RH of 70%, and a photoperiod of 14L:10D.

### 2.2. General Experimental Design

Pilot trials were conducted, followed by eight laboratory bioassays and three field experiments. All experiments commenced when plants were in the mid-whorl stage, four weeks after seedling emergence. Plants were artificially inoculated with FAW larvae in all experiments using a fine camelhair brush. Where relevant, plants were checked for feeding damage to confirm the successful establishment of larvae before applying treatments.

The soil used for application into maize whorls was collected from a maize field at the North-West University, Potchefstroom, South Africa. Other reports also indicated that farmers use soil obtained from the rhizosphere of plants to pour into the maize whorls to control FAW [25]. Wood ash was obtained by burning firewood, which included *Eucalyptus* sp. and *Acacia* sp., after which the ash was sieved using a 2 mm aperture sieve to separate the finer particles from other material. This method of preparing wood ash is similar to that used by farmers [25]. Wet wood ash was prepared by mixing wood ash with water, as described below.

Based on pilot trials, the dosage/application rates of the different treatments were as follows: 10 g of dry ash per plant whorl, 30 g of soil per plant whorl, and for the wet ash treatment, 200 g (ash)/1000 mL (water).

### 2.3. Laboratory Bioassays

#### 2.3.1. General Experimental Design

Two maize seeds (hybrid CRN3505) were planted per 5 L plastic pot, which were maintained under greenhouse conditions until experiments commenced. One plant per pot was removed from pots intended for use in laboratory experiments. All experiments comprised three treatments, i.e., control (untreated), dry ash, and wet ash. The experimental designs were completely randomized designs. Pots were placed in rows inside the laboratory with 90 cm between pots. Based on the results of the pilot experiments, the application rates used for the respective treatments in the experiments described below were as follows: dry ash treatments (10 g/whorl), soil treatments (30 g/whorl), and wet ash (200 g/1000 mL water). A 100 mL suspension of wet ash was poured into the whorl of each plant.

Observations were made of larval movement behavior on plants at 0 min, 0.5, 1.5, 2.5, and 4.5 h after inoculation/treatment. Cardboard sheets (70 cm × 50 cm) were covered with yellow sticky trap sheets and served as a catchment surface area beneath the plants to trap ballooning larvae. An incision was made at the center of the cardboard to fit around the stems of plants. Leaves that were hanging outside the catchment surface area were trimmed to ensure that all larvae were trapped within the catchment surface area to prevent larvae from falling onto the ground and would thus have been unnoticed. The number of larvae that ballooned onto sticky traps was recorded by making a mark on the sticky surface where the larva was trapped.

In the laboratory studies, larval survival was calculated using only the number of larvae that remained on plants, excluding those that either left the plant by silking or walking [33]. The percentage larval mortality ascribed to the effect of the treatment inside plant whorls was therefore calculated after subtracting the number of larvae that ballooned/moved off from plants, from the number that was inoculated onto plants. Percentage mortality was calculated as follows: ((Number of dead larvae per plant/actual number of larvae that remained per plant) × 100). Calculating percentage mortality in this way does, however, not account for cannibalism that may happen. Fortunately, the possible effects of cannibalism on the outcomes of this study were expected to be minimal. In the assays with 5th instars, only two larvae were inoculated per plant, and although this does not prevent cannibalism, it reduces the likelihood that this may happen. In the laboratory experiment on preventative control of 1st instars, larvae were present on plants for only 48 h while in the curative control assays with 1st and 5th instars, the experiments lasted only 48–72 h. Cannibalism during the early instars of *S. frugiperda* is very low [34,35,36]. Low incidences of cannibalism were also expected for large larvae at low densities [37], such as those used in this study.

#### 2.3.2. Effect of Preventative Applications on Survival and Behavior of 1st Instar Larvae

The experiment had three treatments: control, dry ash, and wet ash. Each treatment comprised twelve plants, and each plant served as a replicate. Treatments were applied by pouring either dry or wet ash into the whorls prior to inoculation with larvae. Each plant was then inoculated by placing fifty 1st instar larvae into the whorls, above the ash, immediately after treatment. Plants were observed at the time intervals described above, to record the number of ballooning larvae. The number of larvae that were present on the sticky cardboard and surviving larvae per plant were recorded after 48 h. The percentage larval mortality ascribed to the effect of the treatment itself was then calculated as described above.

#### 2.3.3. Effect of Curative Applications of Ash on Survival and Behavior of Larvae

Three experiments were conducted, one each with 1st, 3rd, or 5th instar larvae. Plants were inoculated with either 50 1st instars, four 3rd, or two 5th instars. Each experiment had three treatments: control, dry ash, and wet ash. Each treatment comprised 12 replicates with one plant per replicate. Larvae were allowed 24 h to establish before plants were treated. Observations on larval behavior and ballooning were recorded at similar time intervals as described above. Plants were dissected and the number of surviving larvae per plant were recorded after four days for 3rd instars, and after 48 h for 1st and 5th instars.

#### 2.3.4. Preference Bioassays

The preferences of 1st and 3rd instar larvae for leaf tissue treated with ash and soil were determined in no-choice and two-choice tests. All treatments were replicated four times with 20 petri dishes per replicate. Bioassays were conducted under constant climate conditions at a temperature of 28 ± 1 °C, RH of 70%, and a photoperiod of 14L: 10D. The positions of larvae inside Petri dishes were recorded after 1, 4, and 24 h to determine their responses, following previously described methods [38]. Larvae that were not present on or underneath leaf discs were recorded as no-response and were excluded from the data analysis [38].

The following treatments were evaluated in no-choice tests: control, wet ash, dry ash, and soil. Square leaf cuttings (discs) of 3 cm × 3 cm were cut from maize whorl leaves. Treatments were applied to the discs by rubbing them gently with ash or soil on both sides. The wet ash treatment was applied by dipping leaf discs into the ash solution, after which they were allowed to dry before use. Each leaf disc was attached to the center of a 7 × 6 cm piece of paper by means of paper clips to hold them in place and to prevent them from moving and folding over. Each maize leaf disc was then placed at the center of a petri dish (9 cm in diameter). Five neonate larvae or one 3rd instar larva were placed on top of each leaf disc.

The following treatment combinations were evaluated in two-choice tests: control vs. dry ash, control vs. wet ash, and control vs. soil. Four leaf discs (1.5 × 1.5 cm) were attached with paper clips as described above. Two of the discs were treated with one of the treatments while the other two served as control. Leaf discs of a similar treatment were placed opposite each other in petri dishes. Either five 1st instars or one 3rd instar larva were placed at the center of the Petri dish and observations were made as described above.

### 2.4. Field Experiments

#### General Experimental Design

Two trials were conducted with 1st instar larvae. The experimental designs were randomized complete blocks. Each field trial comprised five rows of maize plus two border rows. Each row was 15 m long, with an inter-row spacing of 90 cm and intra-row spacing of 10 cm. The first and last plants of each row served as border plants and were not inoculated. Treatments within a row were separated by two plants. Maize hybrid CRN3505 was used in these experiments.

In the first trial, the comparative efficacy of curative and preventative control applications was determined. The trial had six treatments. These were: control, dry ash, and soil, applied either before (preventative) or after (curative) larvae were inoculated into the whorls. Each treatment comprised five replicates and each replicate had seven plants. Each plant was inoculated with twenty 1st instar larvae. For preventative treatments, applications were made into whorls, prior to artificial inoculation with larvae. For curative control treatments, inoculation with larvae was followed by the application of treatments into whorls after 24 h.

In the 2nd field trial, the comparative efficacy of wet and dry ash was determined. The trial had six treatments. These were wet ash and dry ash treatment, each applied curatively and preventatively, as well as two control treatments, one each for the curative and preventative treatments. There were five replicates per treatment and each replicate consisted of seven plants.

In both trials, larval feeding damage to the whorl leaves was rated after five days, using the Davis 1–9 scale [39], after which plants were dissected and the number of surviving larvae recorded. 

A field trial was also conducted to determine the efficacy of treatment against 3rd instar larvae. The four treatments were: control, wet ash, dry ash, and soil. Each treatment was replicated five times with seven plants per replicate. Each pot contained only one plant. An open-pollinated variety (Kalahari) was used. Each plant was artificially inoculated with two 3rd instar larvae and treatments were applied after 24 h. Larval survival was recorded five days after treatment.

### 2.5. Statistical Analysis

Percentage larval mortality data in the laboratory studies, the incidence of larval ballooning, larval survival data for all three field experiments, and the mean damage rating score after calculation per plot for each treatment were analyzed by means of one-way ANOVA followed by a Tukey’s post hoc test. The proportions of the 5th instar larvae that survived were calculated, and data were analyzed by means of binomial distribution tests. Bonferroni correction was used to adjust for multi-means comparisons.

Data on larval ballooning behavior in the experiment conducted to determine the efficacy of curative applications against 1st instar larvae were neither normally distributed nor homogenous. Therefore, it was analyzed using the Kruskal–Wallis test.

Larval response data in no-choice tests at 1, 4, and 24 h were expressed as proportions, and data for each test were analyzed by means of binomial distribution tests, followed by Bonferroni correction to adjust for multiple-mean comparisons. The proportion of larvae exhibiting different preferences between and within treatments was also compared over time. Feeding preference responses in the two-choice tests were also analyzed by means of binomial distribution tests, and choices made were tested against a 50% preference ratio.

All analyses were conducted using Statistica™ Version 14.0.0.15 (TIBCO Software, Inc., 2020) [40].

## 3. Results

### 3.1. Laboratory Bioassays

#### Preventative Application of Ash against 1st Instars

Significantly higher mortalities were recorded on plants that received preventative treatments, compared to the control treatment (*p* = 0.003) (Table 1). Although larval mortality was higher in both the wet and dry ash treatments compared to the control, the mortality recorded for the wet ash treatment did not differ significantly from the control treatment. Percentage mortality was significantly higher in the dry ash treatment than the control treatment, but it did not differ between the dry ash (69%) and wet ash (54%) treatments. The larvae that ballooned off from plants ranged from 18 to 21% and did not differ significantly between the control plants and those treated preventatively with dry or wet ash (*p* = 0.75) (Table 1).

### 3.2. The Effect of Curative Applications on 1st, 3rd and 5th Instar Larvae

Curative applications resulted in significantly higher 1st instar mortality in plants treated with wet ash (66%) and dry ash (67%), compared to the control treatment (22%). Larval mortality did not differ between the wet and dry ash treatments (Table 1). The number of 1st instar larvae that ballooned off from plants did not differ between the control and wet-ash-treated plants, but it was significantly lower for dry ash-treated plants (*p* ≤ 0.03) (Table 1).

The efficacy of curative applications against 3rd instar larvae did not differ significantly (*p* = 0.43) between any of the treatments (Table 1). The number of 3rd instar larvae that migrated off from plants ranged from 11 to 19% and did not differ between treatments (*p* = 0.66) (Table 1).

The mean number of 5th instar larvae that died on the untreated plants (0.23), expressed as a proportion of the total number that remained on those plants (after crawling off from plants was taken into account) was significantly (*p* = 0.04) lower than that on the plants treated with dry ash (0.58). There were no significant differences (*p* > 0.05) between the numbers of larvae that survived the two ash treatments, or between the wet ash and control treatments.

### 3.3. Field Experiments

Survival of 1st instar larvae was generally low, with the highest survival recorded on the control plants of the curative treatment (38%) (Table 2). First instar survival in the experiment that compared preventative and curative applications of soil and dry ash ranged from 19 to 38% (Table 2A). Although there were no significant differences (*p* = 0.12) between treatments, survival was always lower in treated than untreated plants. Leaf damage scores were significantly lower in treated plants (*p* ≤ 0.001) with the lowest damage rating (3.26) recorded for the preventative soil treatment (Table 2A). Curative application of soil did not result in significant reductions in larval feeding damage, but significantly lower damage scores were recorded when a preventative soil application was performed (Table 2A).

The efficacy of wet and dry ash for control of 1st instar larvae did not differ between any of the preventative or curative treatments (*p* = 0.396) (Table 2B). Although not significant, the numbers of surviving larvae were always lower on treated plants. Compared to untreated plants, larval feeding damage scores were significantly (*p* ≤ 0.001) lower on plants that received ash treatments (Table 2B).

Survival of 3rd instar larvae ranged between 21 and 34% for the ash and soil treatments and was significantly lower (*p* ≤ 0.001) than on untreated plants (70%) under field conditions (Table 3).

### 3.4. Preference Bioassays

#### 3.4.1. No-Choice Tests

**Figure 1:**
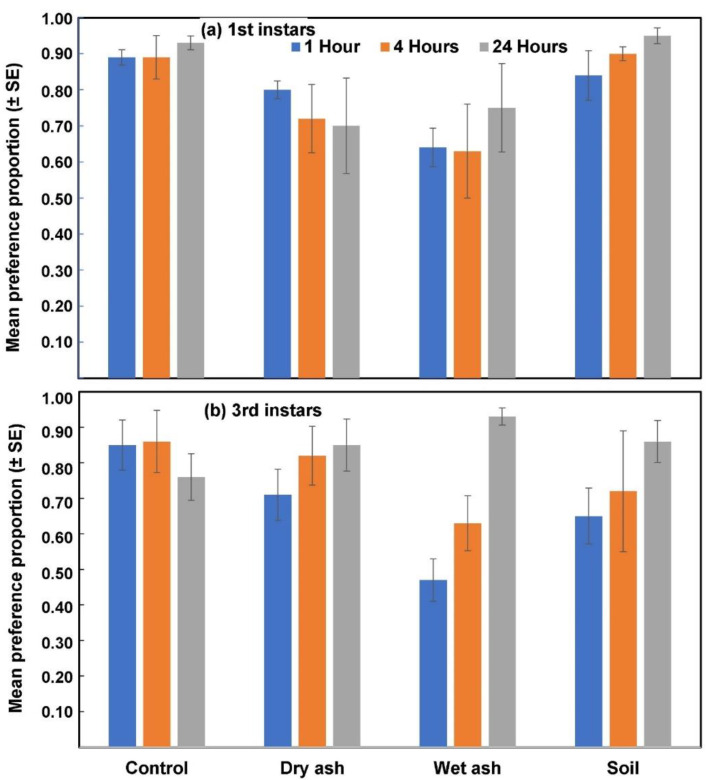
The response of 1st instar (**a**) and 3rd instar (**b**) FAW larvae in no-choice tests, 1, 4 and 24 h after treatment with dry ash, wet ash and soil. Differences between treatments and times are provided in Table 4.

The general tendency was that the initial preference response (1 and 4 h) of 1st instar larvae was markedly positive toward untreated leaves, and it remained high until 24 h after exposure (Figure 1, Table 4). The lowest proportion of 1st instar larvae settled on dry and wet-ash-treated leaves (Figure 1a, Table 4). Preference of 1st instar larvae within each treatment was also compared over time. Results of the binomial distribution tests (Table 4) showed no changes in larval settlement on untreated leaves over time, while those that settled on leaves treated with dry ash decreased significantly over the 24 h period (Figure 1a and Table 4). This was contrary to the response to wet ash and soil treatments, where larval settling on the latter treatments increased significantly over time (Figure 1a and Table 4).

The preference responses of 3rd instar larvae showed that wet-ash-treated leaves were initially (1 h) less preferred compared to the control treatment (Figure 1b, Table 4). Wet-ash-treated leaves were least preferred, followed by those treated with soil (Table 4 and Figure 1b).

#### 3.4.2. Two-Choice Tests

**Figure 2:**
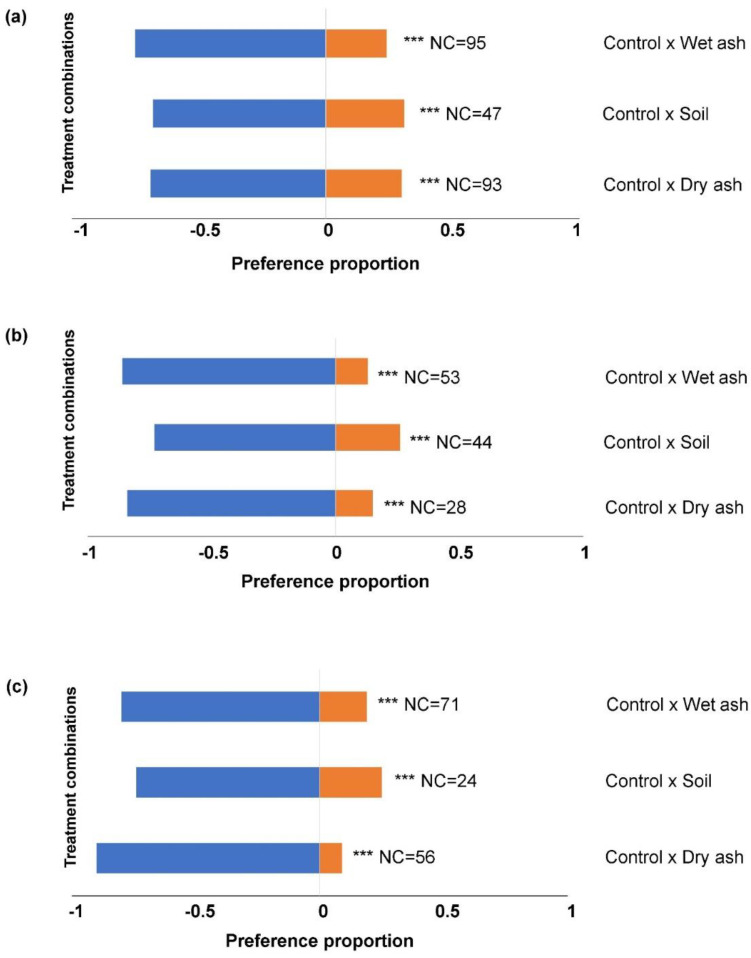
The proportions of 1st instar FAW larvae settling on maize leaf tissue treated with dry ash, wet ash and soil in two-choice tests after, (**a**) 1 h, (**b**) 4 h, and (**c**) 24 h. Significance indicated by *** *p* < 0.001. ‘NC’ indicates the number of larvae (out of 400 per combination) that did not make a choice.

**Figure 3:**
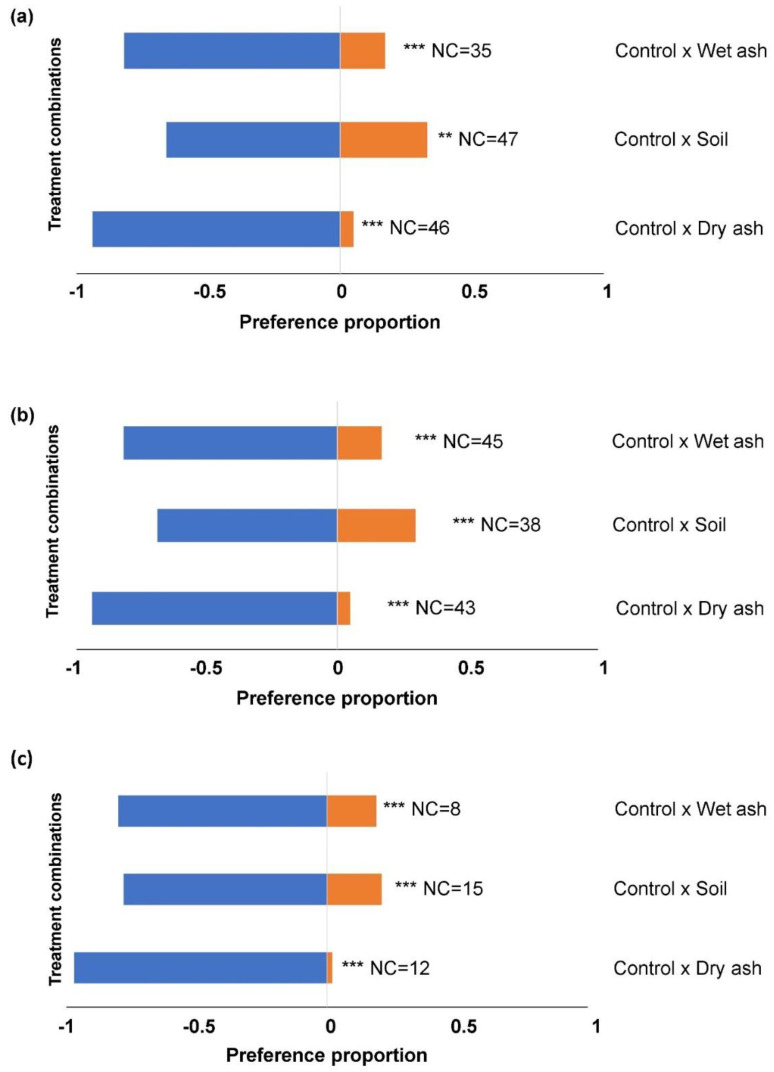
The proportions of 3rd instar FAW larvae settling on maize leaf tissue treated with dry ash, wet ash, and soil in two-choice tests after, (**a**) 1 h, (**b**) 4 h, and (**c**) 24 h. Significance indicated by ** *p* < 0.01, and *** *p* < 0.001. ‘NC’ indicates the number of larvae (out of 80 per combination) that did not make a choice.

The general tendency was that significantly higher numbers of larvae settled on untreated leaf material compared to treated leaves in all the choice-combinations, at all the time intervals (Figure 2 and Figure 3). The number of larvae that did not make a choice between untreated and treated leaves was significantly higher after 1 h (Figure 2a), in contrast to 4 h (Figure 2b) and 24 h (Figure 2c).

## 4. Discussion

In this study, large numbers of larvae moved off from plants, which was evident from the 60–90% reduction in larval numbers from the control plants within a few days after inoculation. Migration and movement away from plants is very common in the Lepidoptera [41]. Most 1st instars of lepidopteran species disappear soon after hatching, and in most cases, these are assumed to be dead. *Spodoptera frugiperda* larvae disperse between plants by means of ballooning. This behavior is not limited to 1st instar larvae and occurs up to the 4th instar [33]. Older larvae disperse by crawling between plants [42]. Larval dispersal to neighboring plants is an important behavioral trait since *S. frugiperda* larvae are cannibalistic and, usually, only one or two larvae per plant develop to the final instar.

It has been reported that between 9 and 96% of 1st instar Lepidoptera larvae get lost in the field [41]. This explains why so many 1st instar larvae disappeared in the field experiments of the present study as opposed to in the laboratory experiments. Predation, cannibalism, dispersal, and weather are some of the mortality factors that impact on numbers of 1st instars [3]. This large reduction in larval numbers in untreated plants complicates the interpretation of results of efficacy studies such as these and limits the use of the Abbot formula [43], which accounts for natural mortality in control treatments [37]. However, the efficacy of control measures against lepidopteran pests is measured in terms of larval survival and feeding damage. The levels of larval ‘mortality’ in plants that received ash or soil treatments in the field studies, may therefore not necessarily be due to the direct killing effect of the ash or soil treatments and could be due to the dispersion of larvae. The efficacy of ash and soil treatments in some of the experiments reported here showed that their application could possibly reduce larval survival and damage. However, the efficacy of these treatments is highly variable.

In this study, whorl application of wet and dry ash resulted in high levels of mortality under laboratory conditions but the effect was much reduced under field conditions. Under laboratory conditions, migration of 1st instar larvae, especially, was limited in ash-treated plants, possibly due to their confinement inside whorls underneath the ash. Under field conditions, none of the preventative or curative applications of soil or ash significantly reduced 1st instar survival, compared to control treatments. Since ballooning by 1st instar larvae is induced or aided by wind [41], the reduced migration observed under laboratory conditions could partly be ascribed to the confined environment with limited air movement. The strong initial non-preference responses of 1st and 3rd instar larvae to treated leaves under laboratory conditions could explain the reduced feeding damage observed in the field trials. These findings are similar to others [44] who reported that maize leaf tissue treated with ash did not exhibit any *S. frugiperda* feeding damage symptoms under laboratory conditions. In this study, no-choice assays with 3rd instars showed that the numbers of larvae that settled on treated leaves increased over time, toward 24 h (Figure 1B). This increase could possibly be ascribed to the effect of starvation of larvae over the 24 h period. The observed non-preference response is therefore not strong enough to prevent larval feeding on treated leaves.

Curative applications effectively reduced 3rd instar survival under field conditions but this was not the case under laboratory conditions. A significant reduction in survival of 3rd instar larvae was recorded under field conditions with dry ash being the most effective. No experiments were conducted with 5th instar larvae under field conditions but laboratory observations showed that larvae succeeded in climbing off from plants by crawling through the ash. Results on the higher mortality of 5th instar larvae on ash-treated plants in the laboratory study were largely inconclusive. The observed mortality cannot be ascribed to only the effect of ash on larvae, since large larvae also exhibit cannibalistic behavior. We did not find any evidence of dead larvae inside the ash when plants were dissected but cannibalism cannot be excluded.

Curative application of dry ash could limit the dispersal of neonate larvae away from natal plants, provided that the application is carried out within a day or two after eggs have hatched. Preventative application of dry and wet ash did not have a significant influence on the migration behavior of 1st instar larvae. However, preventative application of dry ash may limit 1st instar larvae from getting inside the whorl after hatching. The presence of a physical barrier of ash, combined with the non-preference effects of ash and soil may, therefore, provide some protection for maize plants against *S. frugiperda* feeding damage. Sand and soil entrap larvae [45] and limit their access to plant whorls [26,45]. Various studies have reported on the comparative efficacy of dry and wet soil and ash for *S. frugiperda* control [22,46].

Whorl applications of soil, sand, and ash, at 5 g per plant, were reported to provide control of *S. frugiperda* larvae during the vegetative and reproductive plant growth stages of maize [46]. Applications of ash reduced ear damage on maize plants, which received applications of ash at weekly intervals from the V1 stage onward.

A notable difference between this study, which reported variable levels of control, and those that report very good control is the number of applications that were applied. For example, other studies reported at least two applications, but in most cases, weekly applications for up to seven weeks [8,22,46]. A survey of farmers in Uganda showed that the application of ash and soil was often carried out in combination with other methods and that the mean number of management practices per farm ranged from 2.9 to 6.5 [19]. Farmers perceive the efficacy of ash and soil applications against *S. frugiperda* to be high. In Zambia, 54% of farmers considered this method to be effective [23], while between 48.4 and 76.9% of farmers in Ghana, Rwanda, Uganda, Zambia and Zimbabwe perceived it as being effective [12].

### General Observations of Larval Behavior

Larval movement of 1st instars was comparatively more influenced than that of later instars. Visual observations in the laboratory showed differences in behavior of 1st instar larvae on plants that received ash applications either prior to, or after inoculation. When plants were treated preventatively, some larvae dispersed by crawling all over the leaf blades while others were suspended from leaves by means of silk threads. These larvae eventually ballooned off the plants, however, some larvae climbed back onto plants using the silk threads. Similar movement behavior for *S. frugiperda* larvae was reported in other studies [42]. Little larval activity was observed 24 h after treatment/inoculation, as most of the larvae settled on the leaves in all treatments. Some larvae moved to the insides of the whorls of untreated plants and those that received the wet ash treatment since feeding damage was later observed on the whorl leaf tissue. Similar movement behavior of 1st instar larvae, i.e., dispersal within the plants, ballooning, and hanging by silk threads were observed on plants that received the curative and preventive treatments. Curative applications resulted in some larvae moving upward within the whorl after the application. Some 1st instar larvae inside the whorls escaped the dry ash since they were not covered in ash like other larvae. It was however observed that 3rd and 5th instar larvae could crawl through the ash and soil to exit the whorl, prior to feeding on the upper whorl leaves or ballooning off from plants.

Both wet and dry ash did not have a significant impact on the migration behavior of 3rd and 5th instar larvae. Less larval movement was observed on plants treated with dry ash than on untreated or wet-ash-treated plants. It was evident that both these treatments repelled larvae since they were observed climbing upward on the whorl leaves after the application of wet ash, where they remained on the upper part where there was no ash present. In the case of the dry ash treatment, larvae were observed crawling through the dry ash and emerging amongst the whorl leaves after which they would come out of the whorl (escape) and fall onto the sticky trap. This behavior was common in both 3rd and 5th instar larvae. Some of the larvae on ash-treated plants were observed coming out of the whorl through feeding holes in the tightly folded whorl leaves. These larval movement patterns indicated that the non-preference for ash in some way disturbed larvae through repellence. Although large larvae (3rd to 6th instars) usually dwell inside the whorl where they hide and feed, some were observed coming out of the whorl and settling on the leaf blades after the application of ash. This could expose larvae to natural enemies, thereby facilitating biological control.

Since soil and ash carry no potential harm toward the environment recommendations have been made that these control methods be incorporated as elements of IPM programs in smallholder farming systems [22,46,47]. Whorl applications of ash and sand/lime were reported safe for natural enemies such as predatory bugs, earwigs, and lady beetles, in contrast to the application of insecticides such as carbofuran granules [47].

The specific characteristics of soil, sand, and ash that are responsible for causing mortalities of *S. frugiperda* larvae have been described [22,26,46,48]. Sand increases the efficacy of insecticides by enhancing its residual activity inside maize whorls and exerting additional physical effects on the cuticle of larvae since it is in direct contact with larvae inside the whorl, eventually leading to death because of desiccation [45]. Coarse ash may act as a physical mechanism, causing abrasion of the larval cuticle, which leads to desiccation of larvae. The presence of ash on plant leaves could possibly also interfere with the chemical signals emanating from host plants, thus blocking the initial host location by pests. The mechanism through which the microscopic quartz or siliceous particles of sand kill insects is mechanical since these particles penetrate the insect cuticle, creating wounds that lead to loss of body fluid [45]. The mechanism of control provided by the application of sand + lime and soil is described as the abrasion of the soft cuticle of *S. frugiperda* larvae, which eventually leads to death as a result of desiccation [45]. Ash also kills insects by suffocating them as it blocks their spiracles [26].

Soil, ash, and sand do not affect plant growth as they slowly come out of the plant as the plant matures [45]. In addition to the direct physical properties of soil particles, soil often contains a rich diversity of microorganisms that may include both plant- and entomopathogenic organisms. Application of soil into plant whorls may therefore introduce such organisms into plants. While there is no published evidence or reports from farmers about plant diseases following soil application, studies in South America showed very high incidences of entomopathogenic fungi and viruses in soil samples and that these organisms were responsible for the high mortality levels of *S. frugiperda* larvae under laboratory conditions [49,50].

Preventative applications of ash and soil are not a practical option for the control of *S. frugiperda*. Firstly, preventative applications imply whole-field treatment, and secondly, the high labor demands of large-scale use are not cost-effective. There is a risk that the promotion of this approach may result in a substantial increase in the burden of work on women and children, who are often assigned crop-tending tasks [16]. The amount of ash that is needed makes this practice impractical for large-scale preventative control application to all or most plants per field. For example, a single treatment of all the maize plants on a 1 ha field with a plant stand of 60,000, is 300 kg, if applied at the rate used in this study. Nevertheless, using sand and ash remains one of the only tools for *S. frugiperda* control for farmers who cannot afford chemical pesticides [16]. Although these methods are considered environmentally friendly, this benefit would be offset if wood is burnt solely for this purpose.

The variable results obtained in this study should be seen within the context of integrated pest management (IPM) in smallholder maize fields. The efficacy of these methods is influenced by factors such as the plant growth stage at the time of attack, the level of infestation, the size of the larvae, the quality and correct application of the intervention, the use of multiple pest management strategies, climate, and the growing conditions in the field [12,22]. Varying levels of efficacy of these methods between studies can also be ascribed to different types of ash and soils used. However, all soil types are likely to be effective [22] and this will most likely also be the case with ash treatments.

Blanket and vague recommendations on alternative methods such as plant extracts, and ash and soil applications are often made to farmers. These methods do not recognize the needs and expectations of farmers in many cases. What farmers need are methods that can be used to carry out curative control of *S. frugiperda*, once infestations are observed in maize fields. For any treatment to provide effective protection against *S. frugiperda* damage, it needs to provide guaranteed and significant mortality of larvae when applied in the appropriate manner and at the correct time. In the long term, biological control, host plant resistance, and various cultural control practices will reduce pest pressure at regional levels. However, farmers need effective tools to control *S. frugiperda* when infestations are observed at the field level. Recommendations regarding the application of ash and soil should therefore include information on the importance of scouting for early detection of infestations, and specific guidelines on when and how much of these products to apply.

An optimized protocol for using ash and soil applications must consider various factors. The most important of these is the timing of the application [22]. It is therefore recommended that to improve the efficacy of these methods, applications should be made as soon as damage symptoms are observed in maize whorls. Any ash or soil type is likely to have some effect. Regular inspection of fields and early detection of infestation means that larvae are still small and susceptible to treatment. In practice, farmers usually apply ash or soil only into the whorls of damaged plants. Since larvae do migrate between plants, it is further recommended that ash or soil be applied into the whorls of the neighboring plants as well. The application of soil and ash can only be effective during the vegetative stages of crop development when larvae reside in plant whorls, and not when larvae occur on maize ears. This leaves farmers who do not have access to pesticides with no option to control this pest once plants start to flower.

Based on their affordability, environmental friendliness, and possibility to contribute to the suppression of the pest, these methods can be included in IPM programs. The efficacy of these methods may, however, not meet expectations in terms of curative control at the field level.

## 5. Conclusions

The application of soil and ash into maize whorls has the potential to suppress the numbers of early instar larvae but this and other studies showed highly variable results. Since the application of ash and soil has a role in IPM programs for *S. frugiperda*, farmers should be made aware that optimal timing of application is important. Future studies should investigate the effect of two or three applications of soil or ash on larval survival.

## Figures and Tables

**Table 1:** Mean percentage (±SE) mortality and incidence of ballooning of 1st and 3rd instar *Spodoptera frugiperda* larvae on plants treated with ash and soil (laboratory experiment).

Treatment	Mean Larval Mortality (%) ± SE	Mean Incidence of Larval Ballooning (%) ± SE
1st instar: preventative application		
Control	38 ± 4 a *	18 ± 2 a
Wet ash	54 ± 8 ab	20 ± 3 a
Dry ash	69 ± 5 b	21 ± 4 a
1st instar: curative application		
Control	22 ± 4 a	13 ± 3 ab
Wet ash	66 ± 3 b	14 ± 3 a
Dry ash	67 ± 4 b	5 ± 2 b
3rd instar: curative application		
Control	24 ± 6 a	19 ± 7 a
Wet ash	28 ± 7 a	15 ± 7 a
Dry ash	37 ± 8 a	11 ± 5 a

* Means within a column of each section, followed by a different letter differs significantly (*p* < 0.05).

**Table 2:** Mean percentage survival and damage rating scores after preventative and curative applications against of 1st instars, five days after application of treatments under field conditions. Two experiments were conducted: A: Soil and dry ash, B: Wet and dry ash.

Treatment	Mean Larval Survival (% ± SE)	Mean Damage Score (±SE)
**A**: Soil and dry ash		
Curative–control	38 ± 3 a	4 ± 0 c
Curative–dry ash	28 ± 7 a	3 ± 0 ab
Curative–soil	19 ± 3 a	4 ± 0 bc
Preventative–control	26 ± 3 a	4 ± 0 c
Preventative–dry ash	24 ± 5 a	3 ± 0 ab
Preventative–soil	27 ± 6 a	3 ± 0 a
**B**: Wet and dry ash		
Curative–control	13 ± 3 a	4 ± 0 d
Curative–wet ash	7 ± 2 a	3 ± 0 ab
Curative–dry ash	6 ± 2 a	3 ± 0 bc
Preventative–control	11 ± 2 a	4 ± 0 cd
Preventative–wet ash	8 ± 3 a	3 ± 0 ab
Preventative–dry ash	7 ± 4 a	3 ± 0 a

Means within a column followed by the same letter do not differ significantly (*p* > 0.05).

**Table 3:** Mean percentage survival of 3rd instar larvae, five days after curative treatment with soil, wet ash and dry ash (field experiment).

Treatment	Mean Larval Survival (% ± SE)
Control	70 ± 6 a
Wet ash	34 ± 7 b
Soil	25 ± 7 b
Dry ash	21 ± 5 b

Means within a column followed by a different letter differ significantly (*p* < 0.05).

**Table 4:** Results of binomial distribution test of 1st and 3rd instar larval responses after 1, 4, and 24 h in no-choice tests with combinations of different treatments (Bonferroni correction was used).

Treatment	*p*-Value *		
	1 h	4 h	24 h
1st instar larvae			
Control vs. dry ash	<0.001	<0.001	<0.001
Control vs. wet ash	<0.001	<0.001	<0.001
Control vs. soil	0.050	0.640	0.230
Dry ash vs. wet ash	<0.001	0.006	0.130
Dry ash vs. soil	0.141	<0.001	<0.001
Wet ash vs. soil	<0.001	<0.001	<0.001
3rd instar larvae			
Control vs. dry ash	0.030	0.490	0.150
Control vs. wet ash	<0.001	<0.001	0.003
Control vs. soil	0.003	0.030	0.107
Dry ash vs. wet ash	0.002	0.007	0.107
Dry ash vs. soil	0.415	0.131	0.860
Wet ash vs. soil	0.022	0.225	0.148

* Red numbers indicate significant differences. Values < 0.017 are marked in red.

## Data Availability

The datasets used and/or analyzed during the current study are presented in the manuscript.
